# A Potential Biomarker for Predicting Schizophrenia: Metallothionein-1

**DOI:** 10.3390/biomedicines11020590

**Published:** 2023-02-16

**Authors:** Seda Yılmaz, Nülüfer Kılıç, Şüheda Kaya, Gülay Taşcı

**Affiliations:** 1 Department of Psychiatry, Elazığ Fethi Sekin City Hospital, 23100 Elazığ, Turkey; 2 Department of Psychiatry, Elazig Mental Health and Diseases Hospital, 23100 Elazığ, Turkey

**Keywords:** antioxidant, Metallothionein-1, schizophrenia, PANSS, Cu/Zn

## Abstract

It has been thought that oxidative damage may occur in the pathophysiology of schizophrenia; metallothioneins (MT) have strong antioxidant functions. In this study, we aimed to measure MT-1 levels in schizophrenia patients. A total of 52 patients diagnosed with schizophrenia and 38 healthy controls were included in the study. Serum MT-1 concentrations were measured using the Human Metallothionein-1 ELISA Kit. In addition, Cu and Zn levels were measured. PANSS (Positive and Negative Syndrome Scale) was used to determine the disease severity of patients with schizophrenia. The MT-1 levels of the schizophrenia group were lower than the MT-1 levels of the control group. When the correlation analyses were examined, a positive correlation was found between MT-1 and illness duration and Cu/Zn. A negative correlation was found between MT-1 levels and PANSS total scores and PANSS positive scores. In the regression analysis, it was seen that the decrease in MT-1 levels poses a risk for schizophrenia. It was observed that a decrease of 1 ng/mL in MT-1 levels increased the risk of schizophrenia 1.115 times. The low concentration of MT-1 is likely to cause a deficiency in antioxidant defense in patients with schizophrenia. MT-1 may be a useful biomarker for predicting schizophrenia.

## 1. Introduction

Schizophrenia is a disease that usually starts at a young age and has a highly variable clinical course. Many symptoms such as delusions, hallucinations, cognitive impairments, thought disorders, and motor anomalies may be included in the clinical manifestations of this disease. It usually has a progressive clinical course and can cause severe disability [1].

Schizophrenia has attracted people’s attention throughout history and has been the subject of inquiry in many studies conducted so far. The causative factors for the clinical course, the factors that contribute to and exacerbate the disease, have been extensively studied. The factors that cause schizophrenia have always been an especially interesting area of research. However, although many results have been obtained so far, the etiology of schizophrenia has not been fully clarified and enlightened yet. Genetic factors, obstetric anomalies, disruptions in neurotransmitter metabolism, intrauterine infections, abnormal brain imaging findings, and various stress factors have been blamed in studies of the etiology [2]. It has been thought that cognitive deficits occurring at an early age may cause schizophrenia [3]. In some studies, increased ventricular volume and decreased brain volume were observed as neuroimaging findings [4]. In addition, it has been thought that some genes that pose a risk for the development of schizophrenia cause some changes in both cognition and the brain [5]. Some researchers have argued that schizophrenia may result from a brain injury or cerebral defect. However, it has been suggested that brain development is generally normal until clinical symptomatology begins in young adulthood and that schizophrenia may occur later on with a metabolic, infectious, or degenerative factors [6,7]. Neurodegeneration and neuroinflammation play an important role in the pathophysiology of schizophrenia [8] and heavy metals have also been blamed in the etiology in some studies [9]. In addition, it has been thought that oxidative stress may take place in the etiology of schizophrenia and some studies have found that oxidative stress may cause schizophrenia [10].

Metallothionein (MT) was discovered over 60 years ago. It has been the subject of many studies since then. It is an intracellular protein rich in cysteine and with low molecular weight and it has antioxidant properties. MTs have unique structural properties thanks to their strong metal binding and redox capabilities. Binding of MTs to metals occurs through the thiol group in their cysteine residues which are available in their structures. There are 20 cysteine residues that bind to zinc (Zn) [11]. MT-1 and MT-2 are ubiquitous and most commonly expressed isoforms in mammals. They have various functions such as maintaining Zn homeostasis and protection against oxidation and heavy metal damage. They have also very important roles in the metabolism of non-toxic metals such as copper (Cu) and Zn, and toxic heavy metals such as cadmium (Cd) and mercury (Hg) [12]. They are rapidly induced in the liver by drugs, inflammatory mediators, and a wide variety of metals. They show responses according to the status of Zn in the pancreas and intestine. MT-3 is, on the other hand, a brain isoform that has a specific neuronal growth inhibitory activity [13]. MTs also have protective functions against various injuries resulting from reactive nitrogen or oxygen. In other words, MTs are powerful antioxidants [14]. MTs play an important role in the metabolism of Cu and Zn. Abnormalities in Cu and Zn levels have been found in schizophrenia patients [15].

It has been thought that oxidative damage may occur in the pathophysiology of schizophrenia, and as it is known, MTs have strong antioxidant functions. We predicted that one of the causes of oxidative damage in schizophrenia patients might be the deficiency of MTs. There is a growing literature on schizophrenia, but MT-1 levels have not been studied in schizophrenia patients before, and we considered this to be an important knowledge gap. In this study, we aimed to measure MT-1 concentrations from plasma with a practical method that can be easily used in the clinical approach. We examined whether there was a relationship between the MT-1 levels and Cu/Zn, disease severity, and some sociodemographic data. Despite many studies carried out, the factors that cause schizophrenia still remain unclear, and we believe that this study will make important contributions to the etiology and clinical approach of schizophrenia.

## 2. Methods

### 2.1. Study Design

Having previously applied to the outpatient clinics of Elazığ Fethi Sekin City Hospital or being in-patients there, 52 patients diagnosed with schizophrenia according to DSM-V diagnostic criteria and 38 healthy individuals who voluntarily participated in the study were included in this research. Patients with pure schizophrenia were selected according to DSM-V diagnostic criteria. All participants included in the study were between the ages of 18–65. Healthy volunteers without any psychiatric disease history were included in the study as the control group. Exclusion criteria for both the patient and the healthy control group were determined as follows: a history of a neurological disease, the presence of mental retardation, the presence of a disease that would disrupt the metal balance in the body, use of drugs that would disrupt the metal balance in the body (the patient group was under antipsychotic treatment), a history of a chronic disease (excluding schizophrenia history in the patient group), and a history of alcohol or substance abuse. Sociodemographic data were collected from the volunteers and the patients. Demographic data were collected prior to the blood draw with information from the participants. The Positive and Negative Syndrome Scale (PANSS) was administered to the patient group diagnosed with schizophrenia to measure the severity of their disease. All participants were given detailed information about the study and a written consent form was obtained. The study was conducted in accordance with the principles of the Declaration of Helsinki. The study was approved by Elazığ Fırat University Clinical Research Ethics Committee (Date: 10.02.2022, No: 2022/02-33).

### 2.2. Taking Blood Samples from the Patients and Examining the Samples in the Laboratory

#### 2.2.1. Metallothionein-1

In the study, samples were taken from the control and patient groups after 12 h of fasting into a tube containing aprotinin (BD Vacutainer SST II Advance, BD, Plymouth, UK). Blood samples were centrifuged at 2000–3000 rpm for 20 min and plasma containing Metallothionein-1 was placed in small volume tubes for analysis and stored at −20 °C until the study day.

Plasma Metallothionein-1 levels were studied using the Human Metallothionein-1 ELISA Kit (Bioassay Technology Laboratory, catalog no: E4358hu, Shanghai, China) in accordance with the study procedures specified in the kit catalog; absorbance measurement was made using a Chromate 4300 Microplate Reader (Awareness Technology, Palm City, FL, USA) device. The minimum detection limit of Metallothionein-1 was 0.24 ng/mL. The intra-assay and inter-assay coefficient of variation for plasma Metallothionein-1 were <8% and <10%, respectively.

#### 2.2.2. Cu and Zn

Each blood sample (6 mL) was drawn from the antecubital vein. Samples were collected and analyzed in vacuum tubes, including 15% K3 ethylene diamine tetraacetic acid-anticoagulation tubes (Sarstedt, Essen, Belgium). The levels of copper and zinc were tested using the spectrophotometric method on an Abbott Architect c8000 (Abbott, Abbott Park, IL, USA) Chemistry System. The atomic absorption spectroscopy (AAS) method was used for this purpose. AAS is based on the principle of measuring the absorbed amount by the absorption of ultraviolet and visible light by free atoms. The absorbed beam is calculated according to the Lambert–Beer law.

### 2.3. Data Collection Tools

Positive and Negative Syndrome Scale (PANSS)

This scale was developed by Kay et al. in 1987. The Turkish validity and reliability study of the PANSS scale was conducted by Kostakoğlu et al. in 1990. Of the 30 psychiatric parameters evaluated, 7 parameters belong to the positive syndrome subscale, 7 parameters to the negative syndrome subscale, and the other 16 parameters to the general psychopathology subscale. It is a 7-point Likert Scale. Each item is evaluated between 1 and 7. Four measurements are made: positive score, negative score, general psychopathology score, and the PANSS total score. An increase in the scores obtained indicates an increase in the severity of the disease [16,17].

### 2.4. Statistical Analysis

The data obtained in the research were analyzed using the SPSS 22.0 (Statistical Package for Social Sciences) (SPSS Inc., Chicago, IL, USA) for Windows program. Number, percentage, mean, and standard deviation were used as descriptive statistical methods in the evaluation of the data. Differences between the ratios of categorical variables in the independent groups were analyzed with Chi-Square and Fisher’s exact tests. The *t*-test was used to compare quantitative continuous data between the two independent groups. Independent Groups t-Test was performed to determine whether there was a difference in MT-1 levels between patients using one antipsychotic drug and two antipsychotic drugs. Pearson correlation analysis was applied between the continuous variables of the study. Binary Logistic Regression analysis was performed to identify predictive variables for schizophrenia. Linear Regression analysis was performed to determine the predictive parameters of MT-1 levels. Since autocorrelation occurred for the PANSS total in Model-1 Linear Regression analysis, Model-2 Linear Regression analysis was created for the PANSS total. Optimal cut-off values of MT-1 in the presence of schizophrenia were predicted by performing a Receiver-operating characteristic (ROC) analysis. All *p* values were two-tailed and values < 0.05 were considered to indicate statistical significance.

## 3. Results

There was no significant difference between the groups in terms of gender (*X*^2^ = 0.009; *p* = 0.573 > 0.05). There were 42 (80.8%) men and 10 (19.2%) women in the schizophrenia group, and 31 (81.6%) men and 7 (18.4%) women in the control group. There was no significant difference between the groups in terms of age (*p* > 0.05) (Table 1). In the schizophrenia group, it was observed that 11 patients (21.2%) used olanzapine, 11 patients (21.2%) used olanzapine + risperidone, 10 patients (19.2%) used olanzapine + haloperidol, 10 patients (19.2%) used risperidone, and lastly, 10 patients (19.2%) used olanzapine + quetiapine. The mean daily dose of olanzapine was 15 mg, risperidone 2 mg, haloperidol 15 mg, and quetiapine 200 mg. The mean duration of illness in schizophrenia patients was determined as 7.712 ± 5.932 years (Min = 1; Max = 20). There was no significant difference between the two groups regarding smoking (*p* = 0.401) and BMI (body mass index) (*p* = 0.277). The distribution of sociodemographic data of both groups is shown in Table 1.

**Figure 1:**
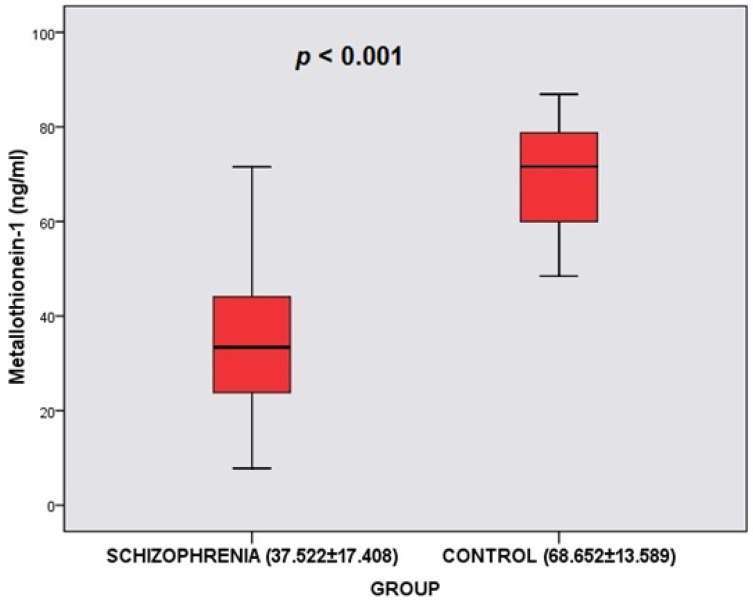
Comparison of Metallothionein-1 between the schizophrenia group and control group.

MT-1 levels differed significantly according to the groups (*t* = −9.165; *p* < 0.001). The MT-1 levels of the schizophrenia group ($\bar{x}$ = 37.522 ng/mL)) were found to be lower than the MT-1 levels of the control group ( $\bar{x}$ = 68.652 ng/mL) (Table 2, Figure 1). Cu levels differed significantly according to the groups (*t* = −3.949; *p* < 0.001). The Cu levels of the schizophrenia group ($\bar{x}$ = 74.906) were found to be lower than the Cu levels of the control group ($\bar{x}$ = 88.934). Zn levels did not differ significantly according to the groups (*p* > 0.05). However, Cu/Zn levels differed significantly according to the groups (*t* = −3.030; *p* = 0.003 < 0.05). The Cu/Zn levels of the schizophrenia group ($\bar{x}$ = 0.938) were found to be lower than Cu/Zn levels of the control group ($\bar{x}$ = 1.125) (Table 2). In the schizophrenia group, it was determined that the PANSS total mean score was 61.058 ± 11.187 (Min = 42; Max = 84), the PANSS positive mean score was 26.404 ± 10.628 (Min = 8; Max = 49), the PANSS negative mean score was 14.519 ± 4.327 (Min = 7; Max = 24), and the PANSS general psychopathology mean score was 20.135 ± 2.635 (Min = 16; Max = 25) (Table 2).

There was no significant difference in MT-1 levels between patients using one antipsychotic drug and two antipsychotic drugs (*t* = 0.312; *p* = 0.752) (Table 3).

**Figure 2:**
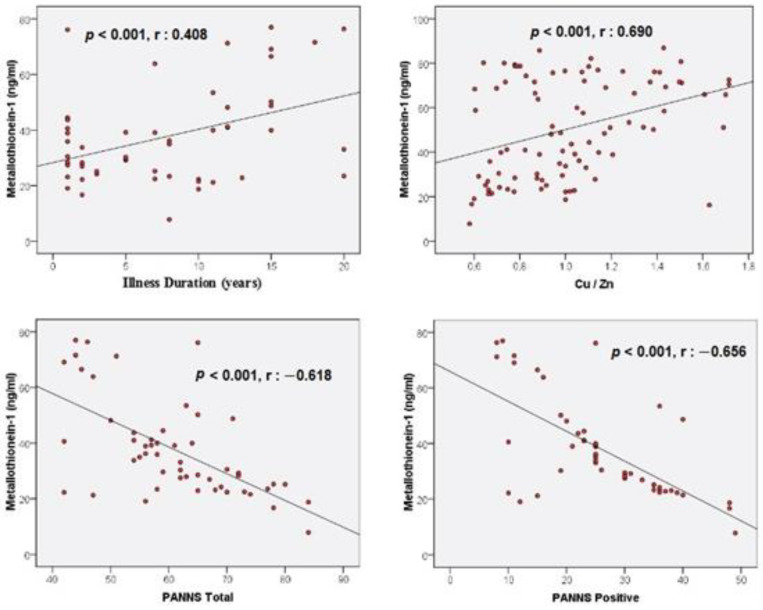
Correlations between illness duration, Cu/Zn, PANSS total, PANSS positive, and Metallothionein-1.

When the correlation analyses were examined, a positive correlation (*r* = 0.408, *p* = 0.003 < 0.05) was found between the illness duration and MT-1 levels (Table 4, Figure 2). A positive correlation (*r* = 0.690, *p* < 0.001) was found between Cu/Zn and MT-1 levels (Table 4, Figure 2). In addition, a negative correlation (*r* = −0.618, *p* < 0.001) was found between the PANSS total scores and MT-1 levels. A negative correlation (*r* = −0.656, *p* < 0.001) was also found between the PANSS positive scores and MT-1 levels (Table 4, Figure 2). Correlations between other variables were not statistically significant (*p* > 0.05) (Table 4).

Binary Logistic Regression analysis, which was established to determine whether independent variables are effective in predicting schizophrenia, is significant and the explanatory value of these variables in predicting schizophrenia is 71.1% (Nagelkerke *R*^2^ = 0.711; *p* < 0.001). It was observed that a decrease of 1 ng/mL in MT-1 levels increased the risk of schizophrenia 1.115 times (1/0.897) (Table 5).

When the Model-1 Linear Regression analysis is examined, it is seen that this model is significant and the MT-1 explanatory value of the variables in the model is 74.5% (*R*^2^ = 0.745; *p* < 0.001). An increase of 0.773 points in the PANSS positive scale is associated with a 1 ng/mL decrease in MT-1 levels (Table 6).

When the Model-2 Linear Regression analysis is examined, it is seen that this model is significant and the MT-1 explanatory value of the variables in the model is 68.5% (*R*^2^ = 0.685; *p* < 0.001). An increase of 0.574 points in the PANSS total scale is associated with a 1 ng/mL decrease in MT-1 levels (Table 7).

**Figure 3:**
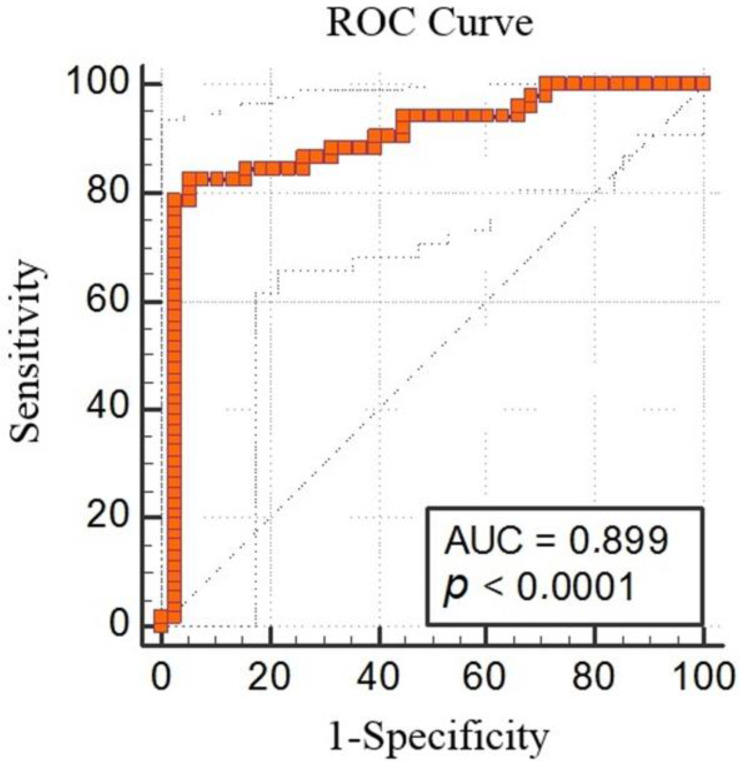
Metallothionein-1 receiver-operating characteristic (ROC) analysis between the schizophrenia group and control group (AUC 0.899, % 95 CI: 0.818, 0.953; *p* < 0.0001; cut off < 50.18).

The ROC analysis demonstrated that MT-1 < 50.18 had 82.69% sensitivity and 94.74% specificity for predicting schizophrenia (AUC: 0.899, % 95 CI: 0.818, 0.953; *p* < 0.0001; cut off < 50.18) (Figure 3).

## 4. Discussion

According to the results of our study, in which we investigated MT-1 concentrations in schizophrenia patients, MT-1 levels in the schizophrenia group were significantly lower than those in the healthy controls. In addition, there was a positive correlation between MT-1 and illness duration and Cu/Zn ratio; furthermore, there was a negative correlation between MT-1 and the PANSS total and PANSS positive scores. In the regression analysis, it was seen that the decrease in MT-1 levels poses a risk for schizophrenia. The increase in the PANSS total and PANSS positive scores was associated with a decrease in MT-1 levels.

Many studies have been conducted on oxidative stress in schizophrenia and a great deal of evidence increasingly supporting the idea that oxidative stress is involved in its etiology has been found. Although some studies do not have consistent results, there are other studies showing a decrease in antioxidant enzyme levels in schizophrenia patients. Compared to controls, reduced glutathione (GSHr), catalase (CAT), and superoxide dismutase (SOD) levels were found to be significantly lower in schizophrenia patients [18]. In another study, SOD and glutathione peroxidase (GSHPx) levels were found to be lower in schizophrenia patients [19]. In a meta-analysis, it was shown that thiobarbituric reactive substances (TBARS) and nitric oxide (NO) as oxidative stress markers increased significantly in schizophrenia patients; therefore, it was observed that oxidative stress was involved in the pathophysiology of schizophrenia [20]. In the brains of post-mortem schizophrenia patients, GSHPx, glutathione (GSH), and glutathione reductase (GR) were found to be quite low in the caudate nucleus [21]. Plasma antioxidants such as albumin, bilirubin, and uric acid were found to be quite low in schizophrenia patients compared to controls [22].

As can be seen, many studies show that oxidative damage may occur in schizophrenia patients and that antioxidant mechanisms may be impaired. MT-1, MT-2, and MT-3 can be found in the brain. Although there is no obvious difference between the amounts of these MTs, the amount of their expression at the mRNA level is as follows: MT-1 (100%), MT-2 (70%), MT-3 (50%) [23]. In this study, we measured the levels of MT-1, which is thought to be the most expressed MT. We obtained an important result confirming our prediction and we saw that MT-1 levels were quite low in schizophrenia patients compared to the controls. The possible reason why MT-1 levels are so low in schizophrenia patients may be insufficient or defective production of MT-1 or a mechanism that interferes with the production process.

MT plays a vital role in the antioxidant defense system; it is a protective protein against ROS damage. Thiolate ligands on cysteine residues provide the redox activity of MT. These cysteine residues can be oxidized by oxidants, and when such a process occurs, Zn is released into the environment. Thus, a decrease in lipid peroxidation products occurs [24]. In cases where there is an increase in oxidative stress in the body, MTs can scavenge many ROS such as reactive nitrogen radicals, nitric oxide radicals, hydrogen peroxide, hydroxyl radicals, and superoxide anions [25]. Free-radical-mediated neuron damage in the central nervous system (CNS) is thought to be involved in the pathophysiology of schizophrenia. As it is known, cellular damage occurs when the antioxidant mechanism is disrupted or the amount of free radicals (oxidoradicals such as superoxide, nitric oxide, hydroxyl ions) increases. There seems to be both an increase in free radicals and a disruption of the antioxidant mechanism in schizophrenia [26]. We think that one of the most important reasons for this insufficient antioxidant defense power may be MT-1 deficiency. Because when compared to GSHPx, SOD, and CAT, MT can be considered as a more effective antioxidant [11]. In addition, the ability of MT to capture hydroxyl ions is 300 times higher than GSH [23]. Because MT has such a strong antioxidant effect, there is a strong possibility that the main defect is the antioxidant activity becomes insufficient due to low MT-1 levels.

Neurodegeneration and neuroinflammation play an important role in the pathophysiology of schizophrenia [8]. Together with an increase in ROS, neuroinflammation causes neuronal dysfunction and damage. In addition, together with macrophages and microglia, reactive astrocytes cause an increase in neuroinflammation and oxidative stress [27]. MT regulates the expression of inflammatory markers in astrocytes, especially TNF-α, IL-3, and IL-6. IL-6 is one of the most important cytokines whose release increases during a brain injury [28]. IL-6 levels are high in schizophrenia patients [29]. MT-1 suppresses the release of proinflammatory cytokines such as IL-6 [30]. In other words, apart from the strong antioxidant activity of MT-1, it also has an anti-inflammatory role. Therefore, the possibility that a pathology in the inflammatory process is related to MT-1 deficiency also comes to mind.

There are animal studies and postmortem studies in the limited research conducted on MT in schizophrenia and psychosis. In a study conducted on rats with a diagnosis of schizophrenia, it was observed that zinc, nitric oxide, and MT levels increased in the ventral hippocampus, and MT levels decreased in the prefrontal cortex in the pre-adolescent period. In the post-adolescent age, increased zinc and nitric oxide and decreased MT levels were observed in the prefrontal cortex. Results obtained after adolescence showed changes in the prefrontal cortex, which is one of the brain regions that are mainly affected in schizophrenia [31]. In a study conducted on postmortem subjects with and without psychosis, up-regulation in MT1E, MT1F, MT1H, MT1K, MT1X, MT2A, and MT3 genes was observed in the dorsolateral prefrontal cortex [32]. The reason for the increase in MT-1 gene expression in that study may be that low MT-1 levels—as we found in our study—might have increased gene expression.

The concentration of MT mainly depends on the state of Cu and Zn [11]. Therefore, we measured Cu and Zn levels, as well as MT-1 levels, to support the results of the study. Previous studies have found conflicting results regarding Cu and Zn levels in schizophrenia [15,33,34,35]. In the results of this study, Cu levels of schizophrenia patients were lower than those of the controls. There was no significant difference between the groups in terms of the Zn levels. However, the Cu/Zn ratio was lower in schizophrenia patients compared to that in controls. Cu/Zn was thought of, and used as, a good indicator in some diseases [36], therefore the Cu/Zn ratio was used in this study. When we examined the correlation between MT-1 level and the Cu/Zn level, we found that there was a positive correlation. As expected, the decrease in the Cu/Zn ratio was associated with a lower MT-1 concentration. Furthermore, in a previous study carried out, it was observed that MT-1 gene transcription increased in the kidneys and livers of mice treated with Hg, Cd, Cu, and Zn [37]. Due to the low Cu and Zn levels in our study, the production of MT-1, which was already produced in low amounts, might not have been induced.

MT mRNA gene expression first increases with age and then decreases with advanced age [38]. In this study, MT-1 levels in schizophrenia patients did not have a significant relationship with age. It is known that exposure to cigarettes causes an inflammatory response, and chronic cigarette smoking may cause a decrease in MT levels by impairing antioxidant activity [39]. However, in the regression analysis performed in our study, it was seen that smoking was not associated with MT-1 levels in schizophrenia patients. In the correlation analysis, it was observed that the MT-1 levels of schizophrenic patients with longer disease duration were higher. We think that this result may be due to the antioxidant activity increased by the antipsychotic medications used in the course of the treatment; the longer the illness duration is, the longer the patients use antipsychotic medications. In previous studies carried out, SOD and CAT activities in schizophrenia patients were found to be higher in those treated than in those untreated [18]. Additionally, SOD levels were higher in patients with chronic schizophrenia than in patients with first episode schizophrenia. This suggested that neuroleptics might promote antioxidant activity [40]. According to the PANSS we used to determine the severity of the disease, there was a negative correlation between the PANSS total and PANSS positive scores and MT-1 levels. In addition, the increase in the PANSS total and PANSS positive scores was associated with a decrease in MT-1 levels. It can be thought that the affected brain regions of patients with positive symptoms are affected more by low MT-1 levels. In addition, in this study, it was found that MT-1 levels were not associated with the different antipsychotic drugs used by the patients.

Antioxidant therapies have a potential for curing, delaying, and preventing the disease in many neurological and psychiatric illnesses including schizophrenia. Treatment with antioxidants may be a good therapeutic strategy for schizophrenia. It has been observed that GSH levels increase in schizophrenia patients using N-acetylcysteine, and various studies have also demonstrated that supplements such as ascorbic acid, α-tocopherol, omega-3, and ginkgo biloba extract have positive effects on schizophrenia patients and cause a decrease in the PANSS scores. [40]. Anti-inflammatory effects have emerged with recombinant MT-1 applied in osteoarthritis patients and positive results have been obtained [30]. We think that adding MT-1 to the treatment of schizophrenia may be a potentially useful treatment in the future.

There are some limitations of our study. The study was carried out with a small group of participants. In order to generalize our results, it would be beneficial to conduct new studies in a larger patient group. Since MT-1 is the most expressed MT, MT-1 levels were measured in this study. Studies involving MT-2, which has a similar effect capacity, can be planned. MT-1 is a protein with many potentials, including antioxidant mechanism, anti-inflammatory effects, and prevention of heavy metal toxicity [41]. It remains unclear what role MT-1 might play in causing or exacerbating schizophrenia. Therefore, in order to increase sufficient evidence, further studies should be conducted by touching upon inflammatory mediators such as cytokines, prooxidants resulting in oxidative stress, and heavy metals together with MT-1 in schizophrenia patients. Many modern methods are available for detecting and diagnosing diseases [42,43,44,45,46,47,48,49,50]. Combining the MT-1 level we have detected in the blood with modern methods in new studies may be an important scientific development.

## 5. Conclusions

Our study showed that the serum MT-1 concentrations of schizophrenia patients are quite low compared to the healthy controls and a decrease in MT-1 levels poses a risk for schizophrenia. In addition, the increase in the PANSS total and PANSS positive scores was associated with a decrease in MT-1 levels. Apart from its other functions, MT-1 is a very powerful antioxidant. The main pathology caused by its low concentration is likely to cause a deficiency in antioxidant defense for schizophrenia patients. In patients with positive symptoms, shorter illness duration, and low Cu/Zn ratio, it would be especially beneficial to measure their MT-1 concentrations using a practical method as in our study. MT-1 may be a useful biomarker for predicting schizophrenia. Moreover, MT-1 may be a potentially beneficial treatment option for schizophrenia in the future.

## Figures and Tables

**Table 1:** Distribution of Sociodemographic Data.

		Schizophrenia (*n* = 52)		Control (*n* = 38)		*x*^2^ (*p*)
		*n*	(%)	*n*	(%)	
Gender	Male	42	(80.8)	31	(81.6)	0.009 (0.573)
	Female	10	(19.2)	7	(18.4)	
Marital Status	Married	11	(21.2)	19	(50)	8.221 (0.004)
	Single	41	(78.8)	19	(50)	
Educational Status	Illiterate	29	(55.8)	7	(18.4)	12.856 (0.005)
	Primary School	9	(17.3)	13	(34.2)	
	Secondary School	8	(15.4)	11	(28.9)	
	University	6	(11.5)	7	(18.4)	
Smoking	Present	38	(73.1)	26	(68.4)	0.232 (0.401)
	None	14	(26.9)	12	(31.6)	
Antipsychotic Drugs Used	Olanzapine	11	(21.2)	-	-	-
	Olanzapine + risperidone	11	(21.2)	-	-	
	Olanzapine + haloperidol	10	(19.2)	-	-	
	Risperidone	10	(19.2)	-	-	
	Olanzapine + quetiapine	10	(19.2)	-	-	
		Mean ± SD		Mean ± SD		*t* (*p*)
Age		36.135 ± 9.826		34.737 ± 9.246		0.683 (0.496)
BMI (kg/m^2^)		26.211 ± 4.006		25.315 ± 3.595		1.093 (0.277)
Illness Duration (years)		7.712 ± 5.932		-		-

Chi-Square Analysis; Independent Groups t-Test. BMI: body mass index.

**Table 2:** Differentiations of Metallothionein-1, Cu, Zn, and Cu/Zn Levels between the Groups and Means of PANSS Scales.

Groups	Schizophrenia (*n* = 52)	Control (*n* = 38)		*t*	*sd*	*p*
	Mean ± SD	Mean ± SD				
Metallothionein-1 (ng/mL)	37.522 ± 17.408	68.652 ± 13.589		−9.165	88	<0.001
Cu (µg/dL)	74.906 ± 13.887	88.934 ± 19.828		−3.949	88	<0.001
Zn (µg/dL)	81.884 ± 12.330	82.244 ± 14.628		−0.126	88	0.900
Cu/Zn	0.938 ± 0.228	1.125 ± 0.356		−3.030	88	0.003
PANSS total	61.058 ± 11.187	-	-	-	-	-
PANSS positive	26.404 ± 10.628	-	-	-	-	-
PANSS negative	14.519 ± 4.327	-	-	-	-	-
PANSS general psychopathology	20.135 ± 2.635	-	-	-	-	-

Independent Groups *T*-Test. PANSS: Positive and Negative Syndrome Scale.

**Table 3:** MT-1 levels according to drug use.

Groups	One Antipsychotic Drug Users (*n* = 21)	Two Antipsychotic Drugs Users (*n* = 31)	*t*	*p*
	Mean ± SD	Mean ± SD		
Metallothionein-1 (ng/mL)	38.445 ± 16.597	36.896 ± 18.180	0.312	0.752

Independent Groups *T*-Test.

**Table 4:** Correlation Analysis with Metallothionein-1 levels.

	Metallothionein-1 (ng/mL)	
	*r*	*p*
Age	−0.167	0.237
Gender	0.163	0.247
BMI	0.151	0.285
Illness Duration	0.408 **	0.003
Cu/Zn	0.690 **	<0.001
PANSS total	−0.618 **	<0.001
PANSS positive	−0.656 **	<0.001
PANSS negative	0.031	0.829
PANSS general psychopathology	−0.031	0.826

** *p* < 0.001; Pearson Correlation Analysis. PANSS: Positive and Negative Syndrome Scale.

**Table 5:** Binary Logistic Regression analysis for independent variables in predicting schizophrenia.

	*β*	*S.E.*	*p*	*OR*	95% C.I. for *OR*	
					Lower	Upper
Cu/Zn	−0.193	1.309	0.883	0.824	0.063	10.722
Metallothionein-1 (ng/mL)	−0.109	0.024	<0.001	0.897	0.855	0.941
Constant	14.232	7058.684	0.998	1,517,275.075	-	-

Model *p* < 0.001; Nagelkerke *R*^2^ = 0.711

**Table 6:** Linear regression analysis for smoking, antipsychotic drugs used, and PANSS scores (Dependent Variable: Metallothionein-1) (Model 1).

	Unstandardized Coefficients		Standardized Coefficients	*t*	*p*
	*B*	Std. Error	*β*		
(Constant)	15.120	18.745		0.807	0.425
Smoking	8.482	4.925	0.170	1.780	0.095
Olanzapine + Risperidone	0.962	4.723	0.023	0.204	0.840
Olanzapine + Haloperidol	−1.292	4.754	−0.030	−0.272	0.787
Risperidone	2.118	4.863	0.048	0.436	0.666
Olanzapin + Quetiapine	0.310	4.710	0.007	0.066	0.948
PANSS positive	−0.773	0.176	−0.472	−4.403	<0.001
PANSS negative	0.232	0.355	0.058	0.653	0.518
PANSS general psychopathology	−0.645	0.592	−0.098	−1.089	0.283

Model *p* < 0,001; *R*^2^ = 0.745. Among the antipsychotic drugs used, those using olanzapine were taken as reference. PANSS: Positive and Negative Syndrome Scale.

**Table 7:** Linear regression analysis for smoking, antipsychotic drugs used, and PANSS total scores (Dependent Variable: Metallothionein-1) (Model 2).

	Unstandardized Coefficients		Standardized Coefficients	*t*	*p*
	*B*	Std. Error	*β*		
(Constant)	25.709	17.002	-	1.512	0.138
Smoking	8.375	4.814	0.166	1.740	0.090
Olanzapine + Risperidone	−1.055	4.932	−0.025	−0.214	0.832
Olanzapine + Haloperidol	−2.014	5.070	−0.046	−0.397	0.693
Risperidone	0.556	5.052	0.013	0.110	0.913
Olanzapin + Quetiapine	−1.190	5.001	−0.027	−0.238	0.813
PANSS total	−0.574	0.168	−0.369	−3.407	0.002

Model *p* < 0,001; *R*^2^ = 0.745. Among the antipsychotic drugs used, those using olanzapine were taken as reference. PANSS: Positive and Negative Syndrome Scale.

## Data Availability

The data presented in this study are available on request from the corresponding author.
